# EXPLORING THE POTENTIAL OF WEARABLE DEVICES FOR HEALTH MONITORING: A FOCUS ON BLACK OLDER ADULTS

**DOI:** 10.1093/geroni/igac059.1000

**Published:** 2022-12-20

**Authors:** Maurita Harris, Shaunik Kapoor, Wendy Rogers

**Affiliations:** University of Illinois Urbana-Champaign, Champaign, Illinois, United States; skapoo21@illinois.edu, Champaign, Illinois, United States; University of Illinois Urbana Champaign, Champaign, Illinois, United States

## Abstract

Chronic diseases are some of the top conditions leading to death in the United States. Management of these chronic diseases could benefit from monitoring risk factors, such as physical activity. Wearable devices (e.g., Fitbit) have the capability to track and give information to allow the individual to take control of their health. However, wearables are typically advertised to young, physically active, and individuals who belong to racial groups with the highest population. As such, the purpose of this study was to understand Black adults’ opinions and attitudes towards wearable design to support usage, focusing on both users and non-users. Participants were interviewed to explore their wants and needs towards wearables to support usage (e.g., aesthetics) and to overcome barriers (e.g., usability concerns). The findings from the thematic analysis will be presented to illustrate these facilitators and barriers identified by Black adults. These insights can guide future inclusive design.

